# Beyond Hemoglobin Concentration: How Intravascular Volume Measurements Can Advance Our Understanding of High‐Altitude Adaptation

**DOI:** 10.1002/ajhb.70179

**Published:** 2025-12-08

**Authors:** Joshua C. Tremblay, Christoph Siebenmann, Mike Stembridge

**Affiliations:** ^1^ Cardiff School of Sport and Health Sciences Cardiff Metropolitan University Cardiff UK; ^2^ Institute of Mountain Emergency Medicine, Eurac Research Bolzano Italy; ^3^ Hochgebirgsklinik Davos Medicine Campus Davos Davos Switzerland

## Abstract

Hemoglobin concentration is often interpreted as a marker for total hemoglobin mass in studies investigating high‐altitude adaptation. However, hemoglobin concentration is determined by both plasma volume and total hemoglobin mass. Therefore, using hemoglobin concentration as a marker for hemoglobin mass can obscure variation in total hemoglobin mass and/or plasma volume and lead to flawed conclusions about adaptation. In this short commentary, we highlight examples from athletic, clinical and high‐altitude populations and responses to environmental stressors illustrating the dissociations between intravascular volumes and hemoglobin concentration. The reliance on hemoglobin concentration has reinforced the prevailing, but potentially incorrect, interpretation of blunted hypoxia‐induced erythropoiesis in Tibetan and Ethiopian highlanders. We argue that measures of plasma volume and total hemoglobin mass, which can easily be obtained using the carbon monoxide rebreathing technique, provide more physiologically relevant phenotypes. We propose that future genetic and evolutionary studies of high‐altitude adaptation should move beyond hemoglobin concentration and focus on measurements of total hemoglobin mass and intravascular volumes.

Advancements in our understanding of physiological, genetic and epigenetic adaptation in high‐altitude populations over the last decade have had a particular focus on hemoglobin concentration [Hb] (Childebayeva et al. 2025). However, it is unlikely that [Hb] is the direct phenotype under selection, but rather represents the effect of selection on another physiological trait—a notion that has long been speculated but never tested (Storz 2010). [Hb] is the amount of hemoglobin in a given volume of blood, usually expressed as g/dl, and is thus dependent on both the circulating total hemoglobin mass and the absolute plasma volume. Accordingly, variations, or lack thereof, in [Hb] can arise from differences in total hemoglobin mass, plasma volume or both. Despite the dependence of [Hb] on plasma volume, in much of the literature on high‐altitude native populations, differences in [Hb] between populations are readily interpreted as differences in total hemoglobin mass. Herein, we use examples from high‐altitude studies as well as other areas of physiology to explain why using [Hb] (and hematocrit; the percentage of blood volume occupied by red blood cells, see Table 1 for calculations) as a marker for total hemoglobin mass and/or intravascular volumes can be misleading in the context of understanding high‐altitude adaptation and propose the direct measurements as a physiologically more relevant alternative. We further illustrate the dissociations between [Hb] and total hemoglobin mass and intravascular volumes in different populations at high altitude and how [Hb] or hematocrit can mask between‐population and within‐individual differences in total hemoglobin mass and intravascular volumes in Figure 1. The purpose of this commentary is to serve as a catalyst to expand our view of the regulation of [Hb] at high altitude. In doing so, we hope to inform and stimulate future work that includes measurements of intravascular volumes when studying adaptation in high‐altitude populations.

**TABLE 1:** Calculations of hematological parameters.

Hemoglobin concentration = Total hemoglobin mass/total blood volume
Hematocrit = Total red cell volume/total blood volume
Total blood volume = Total red cell volume + plasma volume

**FIGURE 1:**
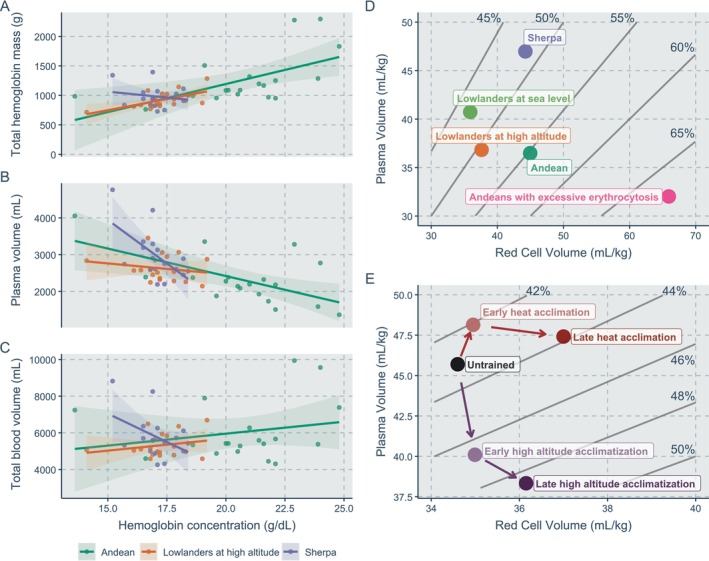
Relationships between hemoglobin concentration ([Hb]) and total hemoglobin mass (panel A), plasma volume (panel B) and total blood volume (panel C) in male Sherpa, Andeans and lowlanders at high altitude (data from (Stembridge et al. 2019)). [Hb] is not related to hemoglobin mass in Sherpa, instead the low [Hb] reflects a higher plasma volume. Note the decoherence between [Hb] and the intravascular volume measures. Panel D presents plasma volume and red cell volume normalized to body mass across male high altitude populations and lowlanders (data from (Stembridge et al. 2019)). Andeans with excessive erythrocytosis were classified as having [Hb] ≥ 21 g/dL. Isolines depict hematocrits. Sherpa hematocrit resembles lowlanders at sea level; however, both plasma and red cell volume are expanded. Note that these plasma volume values differ slightly from Stembridge et al. (2019) because a correction factor for hematocrit (0.91) was not applied here. Panel E presents acclimation/acclimatization to distinct environmental stressors. The reference point reflects the reference values for an untrained male (Oberholzer et al. 2024). Heat acclimation causes an initial increase in plasma volume, followed by erythropoiesis (Jenkins et al. 2025). Conversely, high altitude acclimatization causes plasma volume contraction, followed by erythropoiesis (Siebenmann et al. 2015).

While [Hb] is easily measured, its use as a proxy for total hemoglobin mass can lead to the wrong conclusions. For example, [Hb] is similar among endurance athletes and untrained individuals, even though endurance athletes present a markedly higher total hemoglobin mass that is, however, balanced by an equally expanded plasma volume (Brotherhood et al. 1975; Schmidt and Prommer 2010). Functionally, *total* hemoglobin mass and blood volume are key determinants of cardiorespiratory fitness, defined as maximum oxygen uptake (V̇O_2max_), whereas [Hb] is not (Brotherhood et al. 1975; Schmidt and Prommer 2010). This is because a larger blood volume increases venous return, enabling a higher maximal stroke volume and consequently cardiac output whilst greater total hemoglobin mass maintains the arterial oxygen carrying capacity, together increasing maximal systemic oxygen delivery—but these distinctions are masked if only [Hb] is measured. The decoherence between [Hb] and total hemoglobin mass also occurs in some clinical populations (Otto et al. 2017). Indeed, the assessment of blood volume is critical in understanding the pathophysiology of cardiovascular and renal diseases (Miller 2016; Thijssen et al. 2013). For example, anemia (e.g., low [Hb]) can be due to a decreased hemoglobin mass (true anemia), an expanded plasma volume (dilutional anemia) or a combination of both (Bomholt et al. 2020; Lundby et al. 2018).

This physiological dissociation between [Hb], plasma volume, and total hemoglobin mass becomes particularly evident during environmental adaptations. As low‐altitude residents ascend to high altitude, [Hb] increases. However, initially this increase does not reflect an increase in total hemoglobin mass, but rather a plasma volume contraction (Siebenmann et al. 2015). In a different environmental context, early heat acclimation reduces [Hb] due to an expansion of plasma volume rather than a decrease in total hemoglobin mass. With prolonged heat acclimation [Hb] normalizes, but this occurs as total hemoglobin mass increases while plasma volume remains expanded (Jenkins et al. 2025). These changes, which are masked when only [Hb] or hematocrit are measured, are highlighted in Figure 1E.

A decoherence between [Hb] and total hemoglobin mass can also occur, critically, amongst high‐altitude populations (see Figure 1 panels A–C), since the lower than expected (Hb) in Tibetan highlanders may not reflect a lower total hemoglobin mass, but rather a plasma volume that is larger than in other populations at similar altitudes (Figure 1 panel D) (Stembridge et al. 2019). Despite this, a lower [Hb] in Tibetans and Amharic Ethiopians is widely interpreted as direct evidence for a blunted erythropoietic activity in these populations. While we are not denying that possibility, we are highlighting how [Hb] cannot be used as a marker to test this. Of note, although an expanded plasma volume in Tibetan highlanders has not been confirmed in other studies, Sherpa presented hemoglobin mass expansion akin to lowlanders in response to an extended exposure to 5400 m (Roche et al. 2024) and Tibetans showed no difference in red blood cell volume, despite lower [Hb], compared to Han Chinese in Lhasa (3658 m) (Zhuang et al. 1996). While neither of these studies support a lower total hemoglobin mass at high altitude in Sherpa/Tibetans, they also did not observe the low [Hb] that is widely used as a hallmark of Tibetan high‐altitude adaptation (e.g., 18.2 g/dL in Roche et al. (2024)). Andean populations also present expanded total hemoglobin mass, which is exaggerated in chronic mountain sickness (although not at very high altitude [5100 m]) (Oberholzer et al. 2020) and chronic mountain sickness may be accompanied by a lower plasma volume (Schmidt et al. 2022). Considering the dependence of [Hb] on both plasma volume and total hemoglobin mass, the observation that Tibetan populations do not exhibit lower total hemoglobin mass than lowlanders at high altitude challenges the prevailing dogma (e.g., blunted erythropoiesis) underlying our understanding of Tibetan and Ethiopian high‐altitude adaptation. Future studies thus are required in high‐altitude populations with low [Hb] (e.g., Amharic Ethiopian highlanders) to understand whether this is due to an expanded plasma volume or reduced total hemoglobin mass.

A lower blood viscosity is often used as an explanation for why a low [Hb] is advantageous at high altitude. However, there is considerable nuance in the physiological determinants and functional outcomes of differences in blood viscosity. Blood viscosity is determined by hematocrit, plasma viscosity, red blood cell deformability and red blood cell aggregation. An increase in blood viscosity can increase resistance to blood flow; however, it can also increase shear stress and promote vasodilation (Forconi and Gori 2009). The net resistance to flow as a result of these competing signals depends on the level of blood viscosity and vascular function. Critically, when making inferences from [Hb] or hematocrit, the relationship with blood viscosity is exponential (Pirofsky 1953), particularly in the range of [Hb] measured in individuals with excessive erythrocytosis. Therefore, using [Hb] as a proxy for blood viscosity can be just as vulnerable to misinterpretation as using it as a proxy for total hemoglobin mass.

Collectively, the examples outlined above illustrate that using [Hb] as the phenotype under selection often results in an incomplete or even wrong understanding of physiology—like using body mass index to understand obesity (Ahima and Lazar 2013). It should also be pointed out that there is no need for a proxy since intravascular volumes and total hemoglobin mass can be measured easily and with high precision using the carbon monoxide rebreathing technique (Siebenmann et al. 2017). We thus strongly recommend using this technique to address important open questions, such as the genetic contributions to total hemoglobin mass in different highlander populations. Importantly, blood volume—and its components—are likely more important for selection than [Hb] due to the volumetric effects of pregnancy, where, at sea level, blood volume expands by about 50% via plasma volume expansion and some red cell production (Hytten 1985). Similarly, plasma volume expands by ~40% in high‐altitude residents with Andean and European ancestry (3600 m) (Vargas et al. 2007; Wilson et al. 2007). An inadequate plasma volume expansion during pregnancy is associated with adverse outcomes at sea level (Hays et al. 1985). While less is known at high altitude, in Leadville, USA (3100 m), blood volume expansion during pregnancy was lower in individuals who developed preeclampsia and the blood volume expansion was related to infant birth weight (Zamudio et al. 1993). A promising next step is thus to explore whether natural selection has influenced mechanisms of plasma volume regulation and whether this relates to reproductive success in high‐altitude populations living in their ecological niche. Identifying genetic variants associated with plasma volume (e.g., related to fluid balance, plasma proteins, endothelial cell permeability) would advance understanding of high‐altitude adaptation beyond [Hb].

## Author Contributions

**Joshua C. Tremblay:** conceptualization, writing – original draft preparation, visualization. **Christoph Siebenmann:** writing – review and editing. **Mike Stembridge:** writing – review and editing.

## Ethics Statement

The authors have nothing to report.

## Conflicts of Interest

The authors declare no conflicts of interest.

## Data Availability

Data sharing not applicable to this article as no datasets were generated or analysed during the current study.
